# Persistent Postoperative Chylothorax in a Neonate Undergoing Primary Esophageal Atresia Repair Successfully Treated by Open Thoracic Duct Ligation: A Case Report

**DOI:** 10.7759/cureus.70421

**Published:** 2024-09-29

**Authors:** Zlatan Zvizdic, Alen Pilav, Sabina Terzic, Asmir Jonuzi, Semir Vranić

**Affiliations:** 1 Department of Pediatric Surgery, Clinical Center University of Sarajevo, Sarajevo, BIH; 2 Department of Thoracic Surgery, Clinical Center University of Sarajevo, Sarajevo, BIH; 3 Department of Pathology, Qatar University College of Medicine, Doha, QAT

**Keywords:** chylothorax, esophageal atresia, surgery, thoracic duct ligation, treatment

## Abstract

Chylothorax represents the accumulation of chyle in the pleural cavity due to leakage from the thoracic duct or its tributaries. Intraoperative intrathoracic lymphatic injury is a common cause, but it can also occur on its own. Management of chylothorax involves both medical therapy and, in some cases, surgery for postoperative patients and those who haven't responded to medical therapy. We describe a case of a one-month-old female infant with right-sided chylothorax following primary esophageal atresia repair, who underwent successful thoracic duct ligation by open thoracotomy after unsuccessful medical treatment. Minimally invasive radiology is now the standard treatment for traumatic chylothorax because it is safe and effective. However, surgical ligation of the thoracic duct remains an effective option for treating high-output or recurring chylothorax in countries with limited resources.

## Introduction

Chylothorax occurs when a chyle accumulates in the pleural cavity due to leakage from lymphatic vessels, typically from the thoracic duct. It is the most frequent pleural effusion in neonates. It can be classified based on etiology as congenital (idiopathic), traumatic, and non-traumatic [1]. The incidence of chylothorax in children is lower than in adults [2]. Although chylothorax is most often seen after cardiothoracic surgery (traumatic chylothorax), non-traumatic chylothorax can also occur and is associated with various medical disorders, including malignancies and congenital lymphatic malformations [3]. Untreated, chylothorax is a life-threatening condition associated with high morbidity and mortality because lymph loss can lead to immunosuppression and malnutrition. Therefore, chylothorax requires early diagnosis and adequate management [4].

Biochemical testing of the pleural fluid shows that it contains chylomicrons, high levels of triglycerides (>1.1 mmol/L), an absolute white cell count of more than 1,000/mm³, and lymphocytes (>80%) [5].

Management options include dietary modification and restriction, surgical ligation, and embolization using interventional radiology techniques [2]. Conservative treatment alone is usually sufficient to manage chylothorax. This includes administering somatostatin and octreotide, as well as total parenteral nutrition or medium-chain triglyceride diets [5]. However, in the case of failure, surgical treatment (thoracic duct ligation or pleurodesis) and interventional radiology techniques (thoracic duct embolization) are required [6].

We describe a case of a one-month-old female infant with right-sided chylothorax following primary esophageal atresia repair, who underwent successful thoracic duct ligation by open thoracotomy after unsuccessful medical treatment.

## Case presentation

An 1860-gram newborn girl was delivered by cesarean section at 35 weeks of gestation as the second child in the family. Her family history was unremarkable. The mother was diagnosed with polyhydramnios antenatally. After birth, Apgar scores were 8 and 9 at one and five minutes, respectively. The baby was drooling foamy oral secretions.

**Figure 1:**
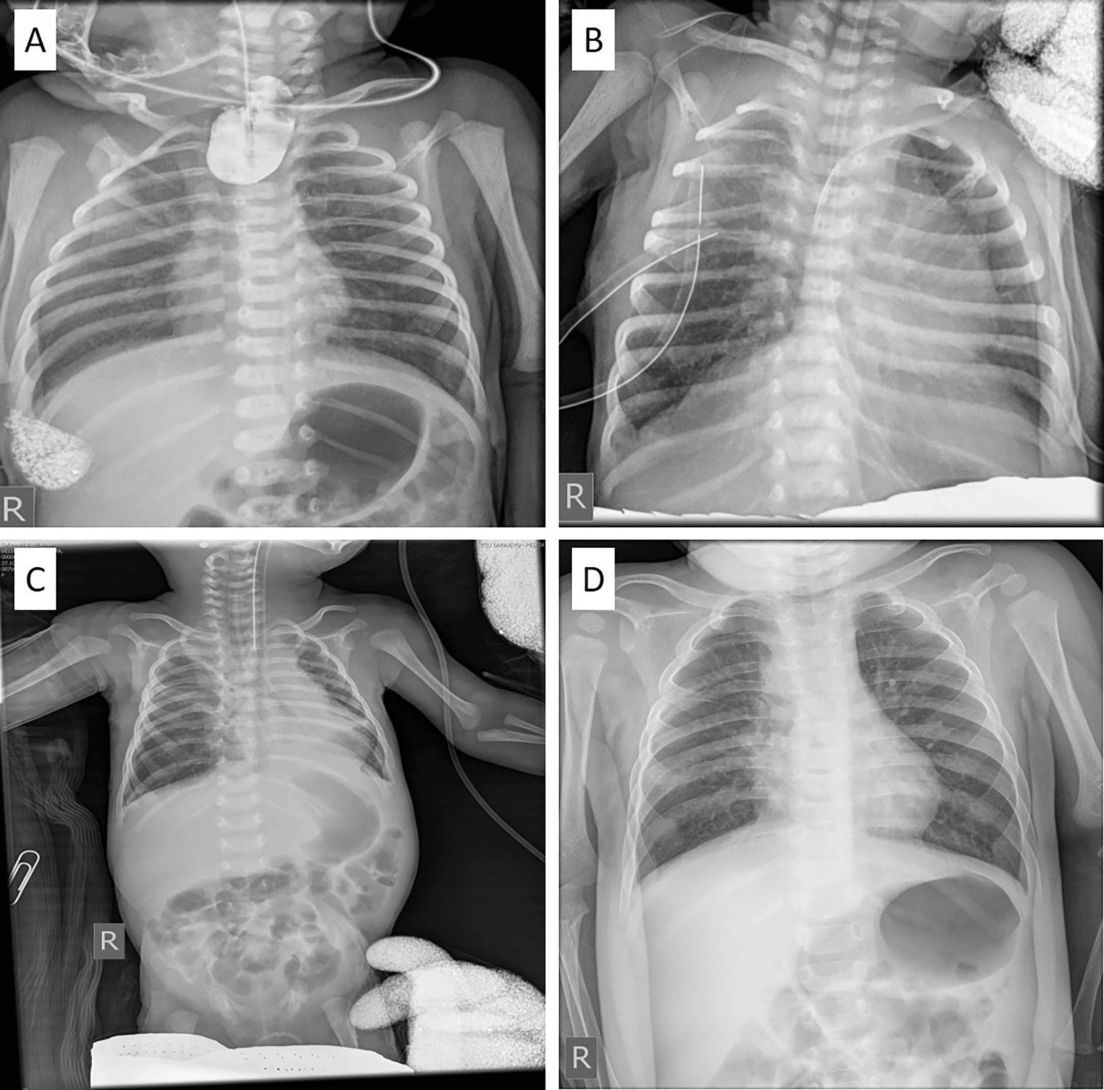
Chest X-rays (A) Preoperative chest X-ray of a neonate showing water-soluble contrast material (iodixanol) in the blind-ending upper esophageal pouch at the third thoracic vertebrae (Th3) with the air in the stomach and intestine; (B) Chest X-ray performed three days after primary esophageal repair showing two chest tubes on the right side for drainage of abundant pleural effusion; (C) Chest X-ray performed two days following open thoracic duct ligation showing the resolution of pleural effusion; (D) Chest X-ray performed seven months after the operation showing bilaterally normal chest finding

Chest and abdominal X-rays showed an orogastric (OG) tube coiling in the upper esophageal pouch, along with a gas-filled stomach and bowel. A clear diagnosis of Gross Type C esophageal atresia was made by injecting a small amount of water-soluble contrast material into the OG tube under the guidance of fluoroscopy (Figure 1A). The assessment involved conducting a cardiac echocardiogram, taking limb and spine X-rays, performing a renal ultrasound, and conducting a comprehensive physical examination of the anus and genitalia to check for any abnormalities; all were unremarkable.

The baby underwent primary repair of esophageal atresia on the second day of life through a right posterior-lateral thoracotomy approach; the incision was made at the level of the fourth intercostal space. The surgery went uneventfully. An intercostal chest tube and a trans-anastomotic tube were finally inserted. After surgery, the baby was returned to the NICU for close monitoring. On the third day of life, the patient developed respiratory distress. A chest X-ray detected the presence of a right pleural effusion, diagnosed through biochemical analysis as chyle. Treatment was initiated by keeping the chest tube in place and placing an additional chest tube on the right side (Figure 1B), along with fasting maintenance and administering parenteral nutrition. Approximately 250-320 mL of chylous drainage was obtained daily. After obtaining informed consent from her parents, we began treatment with subcutaneous octreotide at 4 mg/kg/day on day 5. We proceeded with a gradual increase in the daily dosage and transitioned to a continuous intravenous infusion on the eighth day. Despite reaching the maximum dose of 24 mg/kg/day on day 17, chyle drainage continued unabated for the next week. An indication for operative treatment was set on day 18 because of failed conservative treatment. The patient received 10 mL of oral intralipid through a nasogastric tube four hours before the surgery to aid in spotting the chyle leak and locating the thoracic duct. The operative approach to the chest was trans-pleural. A previously created anastomosis was intact. Lymphatic fluid leakage was detected, originating from the mediastinum beneath the anastomosis. After precise identification of the thoracic duct, the patient underwent ligation through the previous right posterior-lateral thoracotomy in the fourth right intercostal space. The inferior pulmonary ligament was divided, and low ligation of the thoracic duct was performed, with mass ligation of all tissue between the aorta and azygos vein just above the diaphragmatic hiatus. Sutures were placed medially, laterally, and superficially to minimize the risk of esophageal and aortic injuries. In the initial 24 hours, 15 mL of serous fluid was drained by the chest tube, but after that, it remained dry. A chest X-ray revealed both lungs inflated (Figure 1C).

There was no further drainage from the chest tube, and it was removed three days after ligation. Enteral feeding was started on the third postoperative day and gradually increased until full enteral intake was achieved on the seventh day. On day 20, we began reducing the drug dosage and stopped the treatment seven days later (for a total of 22 days of therapy). The follow-up chest X-ray, taken seven months after the operation, was normal (Figure 1D). The pleural effusion did not recur, and the patient was healthy and neurodevelopmentally normal at her 72-month follow-up.

## Discussion

This study described a rare occurrence of chylothorax as a complication of surgery for esophageal atresia, classifying it within the group of postoperative chylothorax. The finding that the chylothorax in our patient was caused by surgical intervention aligns with other studies showing that postoperative chylothorax mostly occurs after thoracic and cardiac surgeries [7,8]. The incidence of chylothorax in neonates is approximately 1 in 24,000 and is estimated to be less than 0.5% following thoracic and cardiac operations [9]. Other causes of chylothorax include congenital lymphatic malformations and malignancies [3]. The appearance of chyle is typically milky, white, and odorless, which, upon standing, forms an immiscible layer at the surface of the fluid. Staats et al. suggested that the most common laboratory tests for detecting chyle should be performed. They indicated that a chylothorax should be suspected if triglyceride levels in the pleural fluid are higher than 1.1 mmol/L and the total cell count exceeds 1,000 cells per mL, with more than 80% lymphocytes [10]. Although chylothorax and pseudochylothorax both typically present a milky white appearance to the pleural fluid, it is essential to distinguish these two entities based on pleural fluid analysis, as they have different pathogenesis and management. Chylothorax is identified by the presence of chylomicrons, a pleural fluid triglyceride level exceeding 110 mg/dL (1.24 mmol/L), and a cholesterol level lower than 200 mg/dL (5.2 mmol/L). In contrast, pseudochylothorax is characterized by exudates with a predominance of neutrophils and a cholesterol level of 200 mg/dL (5.2 mmol/L) or higher, typically with triglyceride levels lower than 50 mg/dL. The biochemical and microscopic analysis of the pleural fluid of our patient met these criteria.

Due to the rarity of this disorder, there is still no clear consensus on its treatment modality. However, Panthongviriyakul and Bines suggested a therapeutic algorithm for managing postoperative chylothorax [11]. As a conservative measure, this includes a diet high in medium-chain triglycerides or total parenteral nutrition, as well as somatostatin or octreotide therapy, surgery, or ensuring proper drainage of the pleural fluid [11]. Adequate replacement of fluids and electrolytes, along with proper nutrition, are fundamental components of conservative treatment. For patients with a high volume of drainage, repeated thoracentesis or continuous drainage is needed. In cases of symptomatic chylothorax, allowing the lung to re-expand is essential for improving pulmonary function [12]. Patients may also benefit from a diet rich in medium-chain triglycerides, as these are absorbed directly into the portal venous system instead of passing through the intestinal lymph vessels and thoracic duct. In most cases, complete parenteral nutrition is also a necessary initial step in conservative treatment. In addition to dietary changes, reduction of lymph flow can be achieved using somatostatin or its analog, octreotide. Both somatostatin and octreotide are considered safe, with rare side effects [13]. However, prospective trials have not confirmed the optimal dosage, method, or duration of drug administration, nor have they established chylothorax as an indication for treatment [13,14]. In our patient, complete parenteral nutrition and treatment with octreotide did not yield any results. Because we could not perform lymphangiography on newborns at our facility, therapeutic thoracic duct embolization was not an option. The only other choice was surgery, which was performed without first identifying the source of the chyle leakage. Various surgical techniques described in the literature could be utilized, including parietal pleurectomy, pleurodesis, ligation of leaking lymphatics, pleuroperitoneal shunts, thoracic duct-venous anastomosis, and ligation of the thoracic duct [15,16]. Lampson [17] was the first to report surgical thoracic duct ligation. Any major surgical procedure performed within eight days of chyle drainage is linked to elevated rates of morbidity and mortality due to a reduction in T lymphocyte count, which impacts the immune system and makes patients more susceptible to infections [18]. Additionally, electrolyte depletion results in metabolic changes, and chylomicrons containing long-chain fatty acids help control these changes [18]. Our patient had no postoperative complications. There is evidence that ligation of the thoracic duct improves collateral lymphatic circulation, regardless of the level of ligation used [19]. This indicates that our patient did not experience any negative effects.

## Conclusions

In conclusion, early diagnosis and proper management of chylothorax are necessary to prevent life-threatening complications resulting from immunosuppression and malnutrition due to lymph loss. The management of chylothorax involves using medical therapy and potentially surgical intervention for postoperative patients and those who have not responded to medical treatment.

Today, minimally invasive radiology is the best way to treat traumatic chylothorax because it is safe and effective. However, surgically ligating the thoracic duct is still an effective option for treating high-output or recurring chylothorax in places with limited resources.
